# Clinical Outcomes of Cardiac Implantable Electronic Device-Related Endocarditis: An International ID-IRI Study

**DOI:** 10.3390/jcm14196816

**Published:** 2025-09-26

**Authors:** Selda Aydin, Ali Mert, Ahmet Naci Emecen, Balint Gergely Szabo, Firdevs Aksoy, Ozay Akyildiz, Sevil Alkan, Antonio Cascio, Oğuz Reşat Sipahi, Botond Lakatos, Muhammed Heja Geçit, Mehmet Emin Bilgin, Şükrü Arslan, Mustafa Yıldız, Zübeyir Bulat, Mehmet E. Gökçe, Fahrettin Katkat, Gülay Okay, Oğuzhan Acet, Serkan Öncü, Selçuk Kaya, Lorenza Guella, Ivica Markota, Juan Pablo Escalera Antezana, Jorge Leonardo Duran Crespo, Abdullah Umut Pekok, Mehmet Ali Tüz, Bilal Ahmad Rahimi, Amani El-Kholy, Hagar Mowafy, Tarsila Vieceli, Edmond Puca, Samir Javadli, Oktay Musayev, Fahad M. Al Majid, Fethi Kılıçarslan, Hakan Erdem

**Affiliations:** 1 Department of Infectious Diseases and Clinical Microbiology, Medical Faculty, Istanbul Medipol University, Istanbul 34214, Türkiye; alimert@medipol.edu.tr; 2 Department of Public Health, Division of Epidemiology, Faculty of Medicine, Dokuz Eylul University, Izmir 35220, Türkiye; ahmet.emecen@deu.edu.tr; 3 National Institute of Hematology and Infectious Diseases, South Pest Central Hospital, 1097 Budapest, Hungary; szabo.balint.gergely@gmail.com (B.G.S.); botondt.lakatos@gmail.com (B.L.); 4 Department of Infectious Diseases & Clinical Microbiology, Faculty of Medicine, Karadeniz Technical University, Trabzon 61080, Türkiye; faslanaksoy@yahoo.com; 5 Department of Infectious Diseases, Adana City Training and Research Hospital, Adana 01230, Türkiye; osaymeclis@yahoo.com; 6 Department of Infectious Diseases and Clinical Microbiology, Onsekiz Mart University School of Medicine, Canakkale 17020, Türkiye; sevil3910@gmail.com (S.A.); eselkaya@gmail.com (S.K.); 7 Department of Sciences for Health Promotion “G. D’Alessandro”, University of Palermo, 90127 Palermo, Italy; antonio.cascio03@unipa.it; 8 A.O.U.P. “Paolo Giaccone”, 90127 Palermo, Italy; 9 Department of Infectious Diseases & Clinical Microbiology, Ege University Faculty of Medicine, Izmir 35040, Türkiye; oguz.resat.sipahi@gmail.com (O.R.S.); oguzhanact@gmail.com (O.A.); 10 Department of Cardiology, Institute of Cardiology, Istanbul University-Cerrahpasa, Istanbul 34098, Türkiye; gecitheja@gmail.com (M.H.G.); mehmeteminbilgin@yandex.com (M.E.B.); sukru.arslan@iuc.edu.tr (Ş.A.); mustafayildiz@yahoo.com (M.Y.); 11 Department of Cardiology, Sirnak State Hospital, Sirnak 73000, Türkiye; zbyrbulat@gmail.com (Z.B.); mehmet_emin_gkc@hotmail.com (M.E.G.); 12 Cardiology Department, Istanbul Training and Research Hospital, Istanbul 34098, Türkiye; fahrettin_katkat@hotmail.com; 13 Department of Infectious Diseases and Clinical Microbiology, Medical Faculty, Bezmialem Vakif University, Istanbul 34093, Türkiye; gulay.okay@hotmail.com; 14 Department of Infectious Diseases and Clinical Microbiology, School of Medicine, Adnan Menderes University, Aydin 09010, Türkiye; droncus@gmail.com; 15 Unità Operativa Malattie Infettive, Ospedale Santa Chiara Trento, 38122 Trento, Italy; lorenza.guella@apss.tn.it; 16 University Clinical Hospital Mostar, 88000 Mostar, Bosnia and Herzegovina; markota.ivica@tel.net.ba; 17 Instituto de Investigaciones Biomédicas e Investigación Social (IIBISMED), Faculty of Medicine, Universidad Mayor de San Simon, Cochabamba P.O. Box 992, Bolivia; jpescaleraa@yahoo.com; 18 Department of Critical & Intensive Care Unit, Hospital Viedma, Cochabamba P.O. Box 591, Bolivia; escacho@gmail.com; 19 Faculty of Medicine, VM Medical Park Pendik Hospital, Istanbul Aydın University, Istanbul 34100, Türkiye; umut.pekok@yahoo.com; 20 Department of Infectious Diseases and Clinical Microbiology, Balikesir University Faculty of Medicine, Balıkesir 10100, Türkiye; mehmetali43@hotmail.com; 21 Department of Pediatrics, Kandahar University Faculty of Medicine, Kandahar 3800, Afghanistan; drbilal77@yahoo.com; 22 Faculty of Medicine, Cairo University, Giza 12613, Egypt; aaakholy@gmail.com (A.E.-K.); hagarmowafy@kasralainy.edu.eg (H.M.); 23 Santa Casa de Porto Alegre, Porto Alegre 90020-090, Brazil; tarsilavieceli@gmail.com; 24 Infectious Diseases Clinic, University Hospital Center “Mother Teresa”, 1001 Tirana, Albania; edmond_puca@yahoo.com; 25 Central Clinic Hospital, Azerbaijan Medical University, Baku 1001, Azerbaijan; scavadov@amu.edu.az (S.J.); dr.oktaymusayev@gmail.com (O.M.); 26 Department of Medicine, Infectious Diseases Division, King Saud University, Riyadh 12372, Saudi Arabia; falmajid@gmail.com; 27 Department of Cardiology, Istanbul Medipol University Medical Faculty, Istanbul 34080, Türkiye; fethi.kilicaslan@medipol.edu.tr; 28 Department of Infectious Diseases & Clinical Microbiology, Gulhane School of Medicine, Turkish Health Sciences University, Ankara 06010, Türkiye; erdemhakan@gmail.com

**Keywords:** endocarditis, cardiac implantable electronic device, transvenous lead extraction, mortality, clinical outcome

## Abstract

**Background/Objectives:** Cardiac implantable electronic device-related infective endocarditis (CIED-RIE) is a serious condition with significant morbidity and mortality. Although recent advances in imaging and therapeutic approaches have improved management, diagnosing and treating CIED-RIE continues to be challenging. This study aimed to identify factors associated with mortality in CIED-RIE patients. **Methods:** We conducted a retrospective, multicenter international study of adult patients diagnosed with CIED-RIE between January 2014 and June 2024. Data on demographics, clinical presentation, microbiological findings, imaging results, treatment modalities, and outcomes were collected and analyzed to determine predictors of short-term mortality. **Results:** A total of 197 patients (mean age: 65.3 ± 14.4 years; 75.1% male) were included. The most common device type was permanent pacemaker (48.2%). Staphylococcus species were the predominant pathogens (62.4%). Surgical intervention was performed in 67.5% of patients, and 90-day mortality occurred in 19.3%. Multivariable analysis identified higher Charlson comorbidity index (HR: 1.31), tricuspid valve involvement (HR: 2.35), vegetation size ≥ 10 mm (HR: 2.53), pulmonary embolism (HR: 3.92), and absence of surgical intervention (HR: 2.90) as independent predictors of increased 90-day mortality. **Conclusions:** Early identification of high-risk patients and prompt multidisciplinary management, including surgical intervention when indicated, are critical to improving outcomes in patients with CIED-RIE.

## 1. Introduction

Cardiac implantable electronic devices (CIEDs), including permanent pacemakers (PPMs), implantable cardioverter–defibrillators (ICDs), and cardiac resynchronization therapy (CRT) devices, are life-saving treatments for various cardiac conditions, used to regulate heart rate and rhythm and to support systolic function. The use of CIEDs has significantly increased over recent decades. This rise is primarily attributed to the aging global population and the broadening of clinical indications requiring device implantation [1]. However, device-related infections remain one of the most serious and challenging complications associated with CIED therapy. CIEDs are recognized as predisposing factors for infective endocarditis [2]. Previous studies have shown that modifiable and non-modifiable factors such as young age, male gender, longer procedure duration, immunosuppression, pocket hematoma, anticoagulant therapy, comorbidities, and device complexity (CRT-D vs. PPM or ICD) contribute to CIED infections [3].

CIED-related infective endocarditis (CIED-RIE) is a serious clinical entity associated with substantial morbidity and mortality, prolonged hospital stays, and increased healthcare costs. Despite recent advances in diagnostic imaging, as well as medical and surgical treatment approaches, CIED-RIE continues to pose diagnostic and therapeutic challenges [4,5]. It is considered the most critical long-term complication of device therapy, significantly complicating management and negatively impacting patient prognosis [6]. A significant increase in the incidence of CIED-RIE has been reported, from 1.7 to 4.8%, along with a decrease in the median age of affected cases. Given these evolving trends, it is likely that clinical outcomes will be significantly influenced.

However, the literature on clinical outcomes and mortality-related factors remains limited, with a few studies involving large cohorts [7,8,9]. Therefore, we aimed to conduct a multicenter, international retrospective study to comprehensively characterize the etiology, treatment approaches, and factors associated with mortality in patients diagnosed with CIED-RIE.

## 2. Materials and Methods

### 2.1. Study Population

This is a retrospective, multicenter study spanning the period between 1 January 2014 and June 2024. The study entailed collecting data from several hospitals with dedicated cardiac units. We included patients with a diagnosis of CIED-RIE and ongoing clinical follow-up at participating centers. The diagnosis of IE was made in accordance with the diagnostic criteria from the 2023 European Society of Cardiology (ESC) Guidelines for Infective Endocarditis and the 2023 Duke-International Society for Cardiovascular Infectious Diseases (ISCVID) Criteria for Infective Endocarditis [2,4].

Inclusion Criteria

- Adults (≥18 years of age)
- Patients with a CIED who present with signs or symptoms suggestive of endocarditis (e.g., fever, chills, new heart murmur, pocket infection, or evidence of infection on imaging) and who have been diagnosed with a definite CIED-RIE.

Exclusion Criteria

- Patients with active infective endocarditis unrelated to a CIED (e.g., native valve endocarditis, prosthetic valve endocarditis) or those with leadless and subcutaneous pacemakers.
- Patients were initially diagnosed according to the diagnostic criteria applied at the respective time and center. All cases were subsequently reassessed using the current diagnostic criteria (2023 ESC and 2023 Duke-ISCVID), and those not fulfilling these definitions were excluded.

### 2.2. Design

The study was designed as a retrospective, international, multicenter investigation and was conducted utilizing the Infectious Diseases—International Research Initiative (ID-IRI) clinical research platform (https://infectdisiri.com/). ID-IRI is a global collaborative organization comprising members from around the world who voluntarily contribute to its research initiatives. Data from patients who met the case definitions were submitted by the centers via a link to a dedicated web page on the Google Forms platform. A total of 27 centers from 11 countries took part in this study, including Türkiye, Saudi Arabia, Italy, Hungary, Egypt, Brazil, Bosnia and Herzegovina, Bolivia, Azerbaijan, Albania, Afghanistan. This study was carried out in accordance with the ethical standards of the institutional research committee and with the 1964 Declaration of Helsinki and its later amendments, or comparable ethical standards. The ethical approval for this study obtained from the Ethics Committee at Istanbul Medipol University (E-10840098-202.3.02-5652; 17 September 2024). However, if the participants are subject to additional ethical requirements mandated by their own institutions, they are responsible for ensuring compliance with those institutional policies and procedures as well.

### 2.3. Data Collection

Data were collected through a web-based questionnaire that included demographic characteristics (age-sex), Charlson comorbidity index (CCI) [10], Pitt bacteremia score [11], type of cardiac implants (PPM, ICD, CRT device), the time interval between the implantation of a CIED and the diagnosis of CIED-RIE (days), the presence of a concurrent or previous pocket infection, a history of CIED-RIE, imaging methods and findings, location and characteristics of vegetation, cardiac complications, the presence of pulmonary septic emboli, acute kidney injury, microbiological data (results of blood culture, pocket site culture, lead culture, serological tests; and monomicrobial or polymicrobial growth), persistent bacteremia, antimicrobial therapy (empirical, targeted, or revised according to culture; and outpatient oral or parenteral therapy after discharge), the need for changes to targeted treatment during follow-up, and the reasons for these changes (allergies, nephrotoxicity, hepatotoxicity, bone marrow suppression, clinical non-response), duration of antimicrobial therapy, the time between the onset of clinical symptoms and appropriate therapy, the presence and type of surgical procedure, interval between diagnosis of CIED-RIE and surgical intervention, the presence and timing of reimplantation, length of hospital stay, survival (at 90 days and one year).

### 2.4. Definitions

CIED-RIE is defined by the presence of a CIED infection accompanied by clinical signs of pocket infection and/or imaging findings (such as lead vegetations or positive FDG-PET on the generator or leads) that meet the criteria for valvular IE according to 2023 ESC, and ISCVID Guidelines for the management of endocarditis [2,4].

The IE timing after CIED implantation: CIED-RIE is temporally classified into early-onset and late-onset forms. Early-onset IE occurs within 12 months of device implantation, while late-onset IE is defined by its onset beyond one-year post-procedure [12].

Definite IE: Two Major criteria, or 1 major criterion and 3 minor criteria, or 5 minor criteria.

The IE-related complications;

Cardiac complications; Heart failure, perivalvular abscess, valvular perforation/rupture, intracardiac fistula, persistent bacteremia.

- Persistent bacteremia is defined as continued positive blood cultures for ≥7 days despite initiation of appropriate antimicrobial therapy [4].
- Heart failure is defined as Ventricular ejection fraction < 50%. https://www.ncbi.nlm.nih.gov/books/NBK553115/, accessed on 22 June 2025.

Pulmonary complications: Pulmonary septic emboli, pneumonia, lung abscess.

Acute Kidney Injury (AKI) was defined according to Kidney Disease Improving Global Outcome criteria as follow; https://www.kidney-international.org/, accessed on 22 June 2025.

Surgical procedures: Valvular repair or replacement, transvenous lead extraction (TLE) or open-heart surgery.

Mortality is defined as death within 90 days following a diagnosis of IE.

### 2.5. Statistical Analysis

Categorical variables were summarized as numbers and percentages, while continuous variables were reported as mean ± standard deviation or median with the 25th–75th percentile, as appropriate. Univariable Cox proportional hazards regression was performed to assess associations with 90-day mortality. Kaplan–Meier survival curves were generated, and differences between groups were evaluated using the log-rank test. Variables that were clinically relevant or statistically significant in univariable analyses were included in the multivariable Cox regression model. Variables considered for the multivariable model with more than 10% missing data were excluded. For the remaining variables in the final model with missing values, imputation was performed using predictive mean matching (PMM). The final model was assessed for multicollinearity and interaction; no violations were identified. All analyses were conducted using R software version 4.3.3 (R Foundation for Statistical Computing, Vienna, Austria; https://www.R-project.org/, accessed on 15 June 2025), and a two-sided *p*-value < 0.05 was considered statistically significant.

## 3. Results

A total of 197 patients diagnosed with CIED-RIE were included in the study, recruited from 27 centers across 11 countries. The distribution of patients by country was as follows: Türkiye (n = 107), Hungary (n = 62), Italy (n = 10), Bolivia (n = 5), Egypt (n = 3), Bosnia and Herzegovina (n = 3), Afghanistan (n = 2), Azerbaijan (n = 2), Saudi Arabia (n = 1), Brazil (n = 1), and Albania (n = 1). The mean age of the patients was 65.3 ± 14.4 years, and 148 (75.1%) were male. Regarding the type of cardiac device, most patients had a PPM (48.2%). Based on the timing of IE onset relative to device implantation, 103 patients (52.3%) had late-onset IE. A previous episode of CIED-RIE was reported in 14 patients (7.11%) (Table 1). The most frequently involved site was the pacemaker leads (68.0%), followed by the right atrium (17.3%), tricuspid valve (14.7%), right ventricle (9.6%), and pulmonary valve (1.5%) (Table 2).

### 3.1. Patients Data

#### 3.1.1. Complications of IE

Heart failure with reduced ejection fraction was observed in 83 patients (42.1%), pulmonary embolism in 39 (19.8%), valvular perforation or rupture in 14 (7.1%), perivalvular abscess in 13 (6.6%), and intracardiac fistula in 7 patients (3.6%). Transthoracic echocardiography (TTE) was the most common diagnostic imaging tool used in 80.7% of cases, followed by Transesophageal echocardiography (TEE) (65%), cardiac magnetic resonance (MR) (3.55%), and Positron emission tomography-computerize tomography (PET-CT) (2.03%).

#### 3.1.2. Microbiological Data

Microbiological evaluation revealed that 156 patients (79.2%) had monomicrobial infections, while polymicrobial growth was detected in 9 patients (4.6%). Blood cultures were positive in 117 patients (59.4%), and persistent bacteremia was identified in 34 of 155 patients with available data (21.9%) (Table 3). The most frequently isolated pathogens were *Staphylococcus* species (62.4%), followed by Gram-negative bacilli (9.6%), *Streptococcus* species (6.6%), *Enterococcus* species (5.6%), and *Candida* species (2.0%). *Staphylococcus aureus*, including both methicillin-susceptible *Staphylococcus aureus* (MSSA) and methicillin-resistant *Staphylococcus aureus* (MRSA) strains, was the most commonly identified organism across all culture sites. MSSA was isolated in 39 blood cultures (19.8%), 31 pocket cultures (15.7%), and 17 lead or vegetation cultures (8.6%). MRSA was also prevalent, found in 23 (11.7%), 19 (9.6%), and 10 (5.1%) of blood, pocket, and lead/vegetation cultures, respectively. *Pseudomonas aeruginosa* (11.1%) was the most frequently isolated Gram-negative bacillus. It was isolated in 12 (6.1%), six (3%) and four (2%) of blood cultures, pocket cultures and lead or vegetation cultures, respectively. 

#### 3.1.3. Antimicrobial Therapy

Antimicrobial treatment was initiated empirically in 179 patients (89.8%). The time interval between the onset of clinical symptoms and the administration of targeted treatment was eight days (interquartile range (IQR): 5–23.8). The survival outcomes did not differ significantly between those who received empirical therapy (81.9% survived vs. 18.1% died) those who received targeted therapy (75.0% vs. 25.0%, *p* = 0.424). Thirty-eight patients underwent treatment modification due to clinical non-response or the occurrence of adverse effects during the follow-up period (13 had clinical non-response, 12 had nephrotoxicity, two patients had an allergy, two patients had bone marrow suppression, two had hepatotoxicity, and seven had other adverse effects). The median duration of antimicrobial therapy was 42.0 days (IQR: 31.5; 50.5). Post-discharge, oral or parenteral antimicrobial therapy was prescribed in 46 (24.2%) and 16 (8.42%) patients, respectively. All of these patients were alive at the end of 90 days.

#### 3.1.4. Surgical Procedures

A total of 133 patients (67.5%) underwent surgery. Open heart operations and valve repairs were performed in 23 cases (17.3%), transvenous lead extraction (TLE) in 104 cases (78.2%), and other surgical methods in six cases (4.5%). The median interval between diagnosis and surgical procedure was 10 days (interquartile range: 7–15). A significant proportion of patients who underwent TLE (89.4%) had subsequently undergone reimplantation. The median time between reimplantation and TLE was 50.0 days (interquartile range: 20.0–72.0).

#### 3.1.5. Outcomes

**Figure 1:**
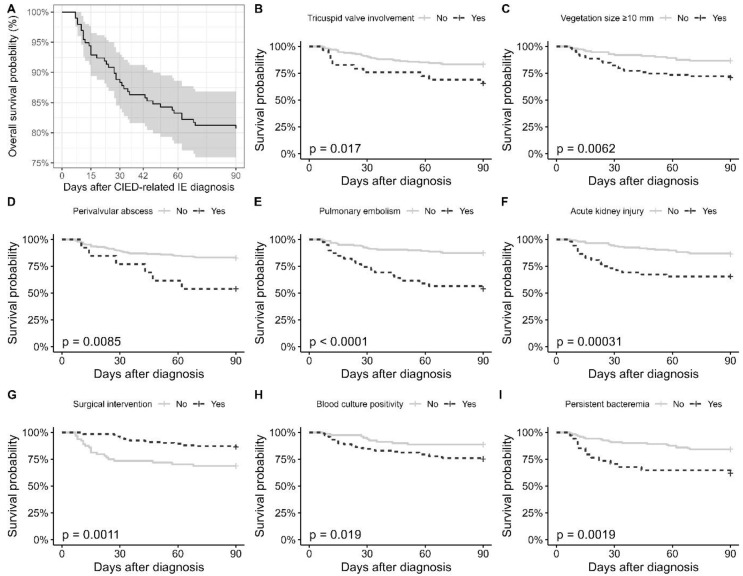
Kaplan–Meier Curves Showing the 90-Day Survival Probabilities in Patients With CIED-Related Infective Endocarditis. (**A**) Overall 90-day survival probability with confidence intervals. (**B**–**I**) Survival curves stratified by variables significantly associated with 90-day mortality.

The mortality occurred in 38 patients (19.3%)**,** with a median time of 27.0 days (range: 7–90) between diagnosis and death. The 90-day survival probability was 80.7% (95% CI: 75.4–86.4%) (Figure 1A). The median length of hospital stay was 43.0 days (IQR: 24.2–56.8). Among the 159 patients who were alive at day 90 and had available follow-up data, 20 (12.6%) died within one year after discharge, 123 (77.4%) were alive, and the outcome was unknown for 16 (10.1%). Approximately one-third of the cases died within one year.

### 3.2. Statistical Results

#### 3.2.1. Univariate Analyses

In univariable Cox regression analysis, higher Charlson comorbidity index (CCI) (HR: 1.25, 95% CI: 1.10–1.42) and Pitt bacteremia score (HR: 1.15, 95% CI: 1.05–1.26) were significantly associated with increased 90-day mortality (Table 1). According to log-rank tests, the 90-day survival probability significantly differed across several clinical variables (Figure 1B–I). Restricted mean survival time (RMST) was significantly lower in patients with tricuspid valve involvement (51.4 ± 6.7 vs. 80.8 ± 2.3 days, *p* < 0.001), acute kidney injury (58.4 ± 4.8 vs. 81.9 ± 2.3 days, *p* < 0.001), and blood culture positivity (62.2 ± 3.0 vs. 85.8 ± 2.4 days, *p* < 0.001). Significant differences were also observed for vegetation size ≥ 10 mm (*p* = 0.002), pulmonary embolism (*p* = 0.002), persistent bacteremia (*p* = 0.002), and perivalvular abscess (*p* = 0.035). Moreover, RMST was significantly lower in patients who did not undergo surgical intervention compared to those who did (68.4 ± 4.2 vs. 83.4 ± 1.6 days, *p* = 0.001), (Table 4).

#### 3.2.2. Multivariate Analyses

**Figure 2:**
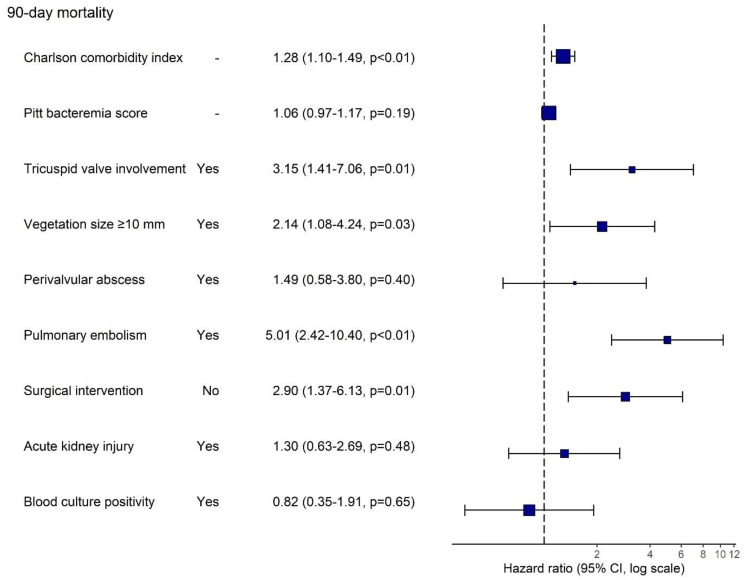
Multivariable Cox Regression Analysis of Factors Associated With 90-Day Mortality in Patients With CIED-Related Infective Endocarditis. Hazard ratios (HR) with 95% confidence intervals are shown on a logarithmic scale for variables included in the multivariable model. Persistent bacteremia was not included in the model due to >10% missing data.

In the multivariable Cox regression analysis, higher CCI (HR: 1.31, 95% CI: 1.13–1.52), tricuspid valve involvement (HR: 2.35, 95% CI: 1.07–5.19), vegetation size ≥ 10 mm (HR: 2.53, 95% CI: 1.28–4.98), pulmonary embolism (HR: 3.92, 95% CI: 1.93–7.99) and absence of surgical intervention (HR: 2.90, 95% CI: 1.37–6.13) remained independently associated with increased 90-day mortality risk (Figure 2).

## 4. Discussion

This study evaluated the etiology, diagnostic and therapeutic approaches, and factors affecting 90-day mortality of CIED-RIE cases. The findings indicated that CIED-RIE remains a complex infection with a high mortality rate. The present study found the overall survival probability to be 80.7% at 90 days in patients with CIED-RIE. Approximately one-third of the cases died within one year. A high Charlson comorbidity index, tricuspid valve involvement, vegetation size ≥ 10 mm, pulmonary embolism and absence of surgical intervention were independent predictors of increased 90-day mortality. These results highlight the significance of specific clinical factors in anticipating adverse outcomes and may aid in risk stratification and clinical decision-making for this high-risk population.

### 4.1. Outcome

Mortality rates reported in previous studies have demonstrated considerable variability, influenced by both the year of data collection and the specific study center. The short-time mortality rate has been reported to range from 4.2% to 36% [13,14,15,16,17] Furthermore, long-term mortality rate has been shown to be as high as 70% [7,9,18]. In our study, the 90-day mortality rate was found to be around 20%. These variable mortality rates may be attributable to variations in institutional experience and the availability of clinical resources across centers.

#### 4.1.1. Comorbidity

The present study demonstrated that a high CCI level was significantly associated with increased mortality in patients diagnosed with CIED-RIE. This finding is consistent with previous studies, which have identified elevated CCI scores as strong predictors of mortality [8,14,19,20]. An elevated comorbidity burden may diminish a patient’s capacity to combat infection, endure prolonged antimicrobial therapy, and surgical intervention [21]. Nevertheless, even in cases where the device is completely and effectively removed, an increased burden of comorbidities has been demonstrated to reduce long-term survival [20]. The association between elevated CCI and mortality underscores the necessity for individualized treatment strategies in patient management and meticulous evaluation of the risks and benefits associated with diverse interventions.

#### 4.1.2. Cardiac Involved and Embolic Events

The development of septic pulmonary emboli in the right-sided infective endocarditis (RSIE) has the potential to cause significant damage by spreading infection to the lung parenchyma or circulatory disturbance. It has been demonstrated that this may result in an exacerbation of right heart failure. It has been reported that the variable most closely associated with embolic complications related to RSIE is vegetation size [22]. In this present study, tricuspid valve involvement, large vegetation and pulmonary embolism were demonstrated to be associated with an elevated mortality risk. It is considered that these findings serve as significant indicators of septic embolization originating from the right heart and subsequent hemodynamic deterioration [13,14,15,20,23]. The results of our study showed that persistent bacteremia is associated with a threefold increase in the risk of mortality. Therefore, we propose that it can be used for risk stratification in clinical practice. Early recognition of these parameters is crucial for the effective management of patients and determining treatment strategies.

#### 4.1.3. Surgery

Surgical intervention was undertaken in two-thirds of our cases, predominantly via TLE. Device reimplantation was performed after a median of 50 days, allowing adequate time for infection clearance. Patients who did not undergo surgery exhibited significantly higher mortality rates, which corroborates existing evidence that surgical management improves in-hospital outcomes [14,20]. Guidelines recommend complete device removal as soon as possible in patients diagnosed with CIED-RIE [1,4,24]. Hence, TLE is a critical component of the CIED-RIE treatment strategy. Despite the well know severity of CIED endocarditis, it has become a safe and feasible procedure [25,26]. However, conservative management may be more appropriate in select patients—such as the elderly, those with high CCI, or at elevated risk for extraction-related complications [20,27,28]. Studies investigating the benefits of TLE demonstrate variable short- and long-term survival data. While some studies report a reduction in in-hospital mortality [18,29], others highlight the advantages of one-year survival [13,30]. Selection of treatment modalities, which depend on the patient, could be the reason for these differences.

#### 4.1.4. The Timing of Infection and Microbiology

In our study, a significant proportion of cases exhibited late-onset infection. However, no association was observed between the timing of infection and mortality, in line with existing evidence [31].

A noteworthy observation was the negative association between pocket infection and mortality. This outcome can be attributed to the role that pocket infection plays in indicating potential clinical complications. The presence of this finding could be considered a clinical sign that facilitates early diagnosis and treatment, rather than one offering direct protection; it triggers further evaluations for potential endocarditis in the patient. This finding is consistent with the lower levels of inflammatory markers, prevalence of vegetation and mortality rates observed in cases of endocarditis with pocket infection, compared to cases with lead related infective endocarditis without pocket infection, as reported in the literature.

On the other hand, it is also probable that this inverse association is due to selection bias resulting from our retrospective study design. Therefore, this finding should be interpreted with caution and confirmed by further studies involving larger patient populations [7].

Previous studies have reported blood culture positivity rates ranging from 65.9% to 86% [9,15,18,32]. In contrast, the present study demonstrated a comparatively lower rate of positive blood cultures. Although blood culture samples were obtained from all patients, the interpretation of this finding is limited by the lack of information regarding prior antibiotic exposure in our data.

Staphylococci species, particularly *S. aureus*, were the predominant pathogens causing CIED-RIE, aligning with previous studies’ findings [14,33,34]. The high virulence and biofilm-forming potential of these bacteria make controlling the infection difficult and necessitate long-term antibiotic treatment.

### 4.2. Limitations

Participation was entirely based on the voluntary initiative of centers, without any probabilistic or systematic sampling strategy. As a result, not all countries within the IDI-IRI network may be represented. This recruitment approach may have introduced selection bias and limited the generalizability of the findings. Additionally, the retrospective design may lead to missing data in some variables. Differences in centers’ experience, resources, and clinical practices may have contributed to heterogeneity across sites, potentially affecting outcomes such as the observed one-year mortality. Finally, our primary focus on 90-day mortality restricts insights into longer-term outcomes. Future prospective studies are warranted to validate these findings and to better characterize long-term prognoses in this complex patient population.

## 5. Conclusions

This multicenter retrospective study underscores the complexity and high mortality associated with CIED-RIE. Our findings emphasize the critical role of specific clinical factors, including a high comorbidity burden, large vegetation size, tricuspid valve involvement, and the presence of pulmonary embolism, as predictors of short-term mortality. Surgical intervention and early recognition of signs like pocket infection were associated with improved outcomes, underscoring the importance of timely and aggressive management. These insights underscore the necessity for individualized treatment strategies and continuous vigilance in the management of CIED-RIE patients to improve survival.

## Figures and Tables

**Table 1:** Demographic and Clinical Characteristics in Relation to 90-Day Mortality.

	All, n = 197	90-Day Mortality		Univariable HR (95% CI)
		Survived, n = 159	Died, n = 38	
Sex				
Female	49 (24.9%)	39 (79.6%)	10 (20.4%)	Ref.
Male	148 (75.1%)	120 (81.1%)	28 (18.9%)	0.94 [0.46; 1.94]
Age, mean ± SD	65.3 ± 14.4	65.0 ± 15.1	66.6 ± 11.0	1.01 [0.99; 1.03]
Charlson comorbidity index, median (25–75%)	4.00 [2.00; 5.00]	4.00 [2.00; 5.00]	5.00 [3.25; 6.00]	1.25 [1.10; 1.42] *
Pitt bacteremia score, n = 184, median (25–75%)	1.00 [0.00; 5.00]	1.00 [0.00; 4.25]	3.00 [1.00; 7.00]	1.15 [1.05; 1.26] *
Type of cardiac implantable device				
CRT	34 (17.3%)	29 (85.2%)	5 (14.7%)	Ref.
ICD	68 (34.5%)	51 (75.0%)	17 (25.0%)	1.86 [0.69; 5.04]
PPM	95 (48.2%)	79 (83.2%)	16 (16.8%)	1.24 [0.46; 3.40]
Timing of IE according to implantation of CIED				
Early onset (<1 year)	94 (47.7%)	73 (77.7%)	21 (22.3%)	Ref.
Late onset (>1 year)	103 (52.3%)	86 (83.5%)	17 (16.5%)	0.72 [0.38; 1.37]
Pocket infection history				
No	158 (80.2%)	126 (79.7%)	32 (20.3%)	Ref.
Yes	39 (19.8%)	33 (84.6%)	6 (15.4%)	0.70 [0.29; 1.67]
Current pocket infection				
No	96 (48.7%)	68 (70.8%)	28 (29.2%)	Ref.
Yes	101 (51.3%)	91 (90.1%)	10 (9.90%)	0.30 [0.14; 0.61] *
History of CIED-related IE				
No	183 (92.9%)	147 (80.3%)	36 (19.7%)	Ref.
Yes	14 (7.11%)	12 (85.7%)	2 (14.3%)	0.70 [0.17; 2.89]

CRT: Cardiac Resynchronization Therapy; ICD: Implantable Cardioverter–Defibrillator; PPM: Permanent Pacemaker. * *p* < 0.05; HR (95% CI): Hazard Ratio with 95% Confidence Interval.

**Table 2:** Anatomical Involvement and Complications in Relation to 90-Day Mortality.

	All, n = 197	90-Day Mortality		HR (95% CI)
		Survived, n = 159	Died, n = 38	
Pacemaker leads involvement				
No	63 (32.0%)	47 (74.6%)	16 (25.4%)	Ref.
Yes	134 (68.0%)	112 (83.6%)	22 (16.4%)	0.64 [0.34; 1.22]
Tricuspid valve involvement				
No	168 (85.3%)	140 (83.3%)	28 (16.7%)	Ref.
Yes	29 (14.7%)	19 (65.5%)	10 (34.5%)	2.39 [1.16; 4.92] *
Pulmonary valve involvement				
No	194 (98.5%)	156 (80.4%)	38 (19.6%)	Ref.
Yes	3 (1.52%)	3 (100%)	0 (0.00%)	-
Right atrium involvement				
No	163 (82.7%)	132 (81.0%)	31 (19.0%)	Ref.
Yes	34 (17.3%)	27 (79.4%)	7 (20.6%)	1.03 [0.45; 2.33]
Right ventricle involvement				
No	178 (90.4%)	144 (80.9%)	34 (19.1%)	Ref.
Yes	19 (9.64%)	15 (78.9%)	4 (21.1%)	1.15 [0.41; 3.23]
Vegetation size ≥ 10 mm, n = 191				
No	112 (58.6%)	97 (86.6%)	15 (13.4%)	Ref.
Yes	79 (41.4%)	56 (70.9%)	23 (29.1%)	2.40 [1.25; 4.59] *
Multiple vegetations, n = 187				
No	134 (71.7%)	106 (79.1%)	28 (20.9%)	Ref.
Yes	53 (28.3%)	44 (83.0%)	9 (17.0%)	0.79 [0.38; 1.68]
Mobile vegetation, n = 184				
No	118 (64.1%)	95 (80.5%)	23 (19.5%)	Ref.
Yes	66 (35.9%)	54 (81.8%)	12 (18.2%)	0.92 [0.46; 1.86]
Heart failure with EF < 50%				
No	114 (57.9%)	95 (83.3%)	19 (16.7%)	Ref.
Yes	83 (42.1%)	64 (77.1%)	19 (22.9%)	1.43 [0.76; 2.70]
Valvular perforation/rupture				
No	183 (92.9%)	148 (80.9%)	35 (19.1%)	Ref.
Yes	14 (7.11%)	11 (78.6%)	3 (21.4%)	1.15 [0.35; 3.75]
Perivalvular abscess				
No	184 (93.4%)	152 (82.6%)	32 (17.4%)	Ref.
Yes	13 (6.60%)	7 (53.8%)	6 (46.2%)	3.05 [1.28; 7.31] *
Intracardiac fistula				
No	190 (96.4%)	155 (81.6%)	35 (18.4%)	Ref.
Yes	7 (3.55%)	4 (57.1%)	3 (42.9%)	2.31 [0.71; 7.52]
Pulmonary embolism				
No	158 (80.2%)	138 (87.3%)	20 (12.7%)	Ref.
Yes	39 (19.8%)	21 (53.8%)	18 (46.2%)	4.47 [2.36; 8.46] *
Acute kidney injury				
No	145 (73.6%)	125 (86.2%)	20 (13.8%)	Ref.
Yes	52 (26.4%)	34 (65.4%)	18 (34.6%)	3.02 [1.60; 5.72] *
Surgical intervention				
No	64 (32.5%)	44 (68.8%)	20 (31.2%)	2.79 [1.48; 5.28] *
Yes	133 (67.5%)	115 (86.5%)	18 (13.5%)	Ref.

EF: Ejection fraction; * *p* < 0.05; HR (95% CI): Hazard Ratio with 95% Confidence Interval. The number of observations is provided only for variables with missing data; all other variables have complete data for all patients.

**Table 3:** Culture Results and Their Associations With 90-Day Mortality.

	All, n = 197	90-Day Mortality		HR (95% CI)
		Survived, n = 159	Died, n = 38	
Any culture positivity				
None	32 (16.2%)	27 (84.4%)	5 (15.6%)	Ref.
Monomicrobial	156 (79.2%)	127 (81.4%)	29 (18.6%)	1.21 [0.47; 3.13]
Polimicrobial	9 (4.57%)	5 (55.6%)	4 (44.4%)	3.76 [1.01; 14.0]
MRSA growth				
No	162 (82.2%)	134 (82.7%)	28 (17.3%)	Ref.
Yes	35 (17.8%)	25 (71.4%)	10 (28.6%)	1.83 [0.89; 3.78]
MRCoNS growth				
No	183 (92.9%)	146 (79.8%)	37 (20.2%)	Ref.
Yes	14 (7.11%)	13 (92.9%)	1 (7.14%)	0.34 [0.05; 2.47]
Gram-negative organism growth				
No	179 (90.9%)	146 (81.6%)	33 (18.4%)	Ref.
Yes	18 (9.14%)	13 (72.2%)	5 (27.8%)	1.68 [0.66; 4.31]
Blood culture positivity				
No	80 (40.6%)	71 (88.8%)	9 (11.2%)	Ref.
Yes	117 (59.4%)	88 (75.2%)	29 (24.8%)	2.42 [1.14; 5.11] *
Pocket culture positivity				
No	126 (64.0%)	96 (76.2%)	30 (23.8%)	Ref.
Yes	71 (36.0%)	63 (88.7%)	8 (11.3%)	0.43 [0.20; 0.93] *
Lead/vegetation culture positivity				
No	149 (75.6%)	116 (77.9%)	33 (22.1%)	Ref.
Yes	48 (24.4%)	43 (89.6%)	5 (10.4%)	0.43 [0.17; 1.11]
Persistent bacteremia, n = 155				
No	121 (78.1%)	102 (84.3%)	19 (15.7%)	Ref.
Yes	34 (21.9%)	21 (61.8%)	13 (38.2%)	2.90 [1.43; 5.89] *

* *p* < 0.05; HR (95% CI): Hazard Ratio with 95% Confidence Interval. MRSA: Methicillin-Resistant *Staphylococcus aureus*; MRCoNS: Methicillin-Resistant Coagulase-Negative *Staphylococci.* The number of observations is provided only for variables with missing data; all other variables have complete data for all patients.

**Table 4:** Restricted Mean Survival Time and Log-Rank Test Results for Variables Significantly Associated With 90-Day Mortality.

		RME (Days) ± SE	Log-Rank *p*-Value
Tricuspid valve involvement	No	80.8 ± 2.3	<0.001
	Yes	51.4 ± 6.7	
Vegetation size ≥ 10 mm	No	82.3 ± 2.2	0.002
	Yes	67.6 ± 4.2	
Perivalvular abscess	No	78.7 ± 2.3	0.035
	Yes	61.4 ± 6.4	
Pulmonary embolism	No	80.4 ± 2.4	0.002
	Yes	61.2 ± 4.9	
Acute kidney injury	No	81.9 ± 2.3	<0.001
	Yes	58.4 ± 4.8	
Surgical intervention	No	68.4 ± 4.2	0.001
	Yes	83.4 ± 1.6	
Blood culture positivity	No	85.8 ± 2.4	<0.001
	Yes	62.2 ± 3.0	
Persistent bacteremia	No	78.9 ± 2.5	0.002
	Yes	59.1 ± 4.9	

RME: Restricted Mean Survival Time; SE: Standard Error.

## Data Availability

The data presented in this study are available on request from the corresponding author due to privacy and ethical restrictions.

## Abbreviations

The following abbreviations are used in this manuscript:

CIEDs	Cardiac implantable electronic devices
PPMs	Permanent pacemakers
ICDs	Implantable cardioverter–defibrillators
CRT	Cardiac resynchronization therapy
CIED-RIE	Cardiac implantable electronic device-related infective endocarditis
ESC	European Society of Cardiology
ISCVID	International Society for Cardiovascular Infectious Diseases
ID-IRI	Infectious Diseases—International Research Initiative
CCI	Charlson comorbidity index
FDG-PET	Fluorodeoxyglucose positron emission tomography
AKI	Acute Kidney Injury
TLE	Transvenous lead extraction
TTE	Transthoracic echocardiography
TEE	Transesophageal echocardiography
MR	Magnetic resonance
PET-CT	Positron emission tomography-computerize tomography
MSSA	Methicillin-susceptible *Staphylococcus aureus*
MRSA	Methicillin-resistant *Staphylococcus aureus*
MRCoNS	Methicillin-Resistant Coagulase-Negative Staphylococci.
IQR	interquartile range
